# Short communication: A33scFv–cytosine deaminase: a recombinant protein construct for antibody-directed enzyme-prodrug therapy

**DOI:** 10.1038/sj.bjc.6600751

**Published:** 2003-03-18

**Authors:** P M Deckert, C Renner, L S Cohen, A Jungbluth, G Ritter, J R Bertino, L J Old, S Welt

**Affiliations:** 1 Medizinische Klinik III, Universitätsklinikum Benjamin Franklin, Freie Universität Berlin, D-12200 Berlin, Germany; 2 Medizinische Klinik I, Universitätskliniken des Saarlandes, D-66421 Homburg/Saar, Germany; 3 Ludwig Institute for Cancer Research – New York Branch, 1275 York Avenue, New York, NY 10021, USA; 4 Molecular Pharmacology, Memorial Sloan-Kettering Cancer Center, 1275 York Avenue, New York, NY 10021, USA

**Keywords:** tumour targeting, A33 antibody, antibody directed enzyme-producing therapy (ADEPT), colon carcinoma, recombinant fusion proteins

## Abstract

A recombinant fusion protein of colon carcinoma binding A33 single chain antibody with cytosine deaminase displayed specific antigen binding and enzyme activity in surface plasmon resonance and is catalytic activity assay. *In vitro*, it selectively increased the toxicity of 5-FC to A33 antigen-positive cells by 300-fold, demonstrating the potency of this ADEPT strategy.

Antibody-directed enzyme-prodrug therapy (ADEPT) utilises antibody–enzyme constructs for targeted enzyme delivery to tumours and subsequent localised activation of a prodrug. Its potential has been demonstrated in phase I studies (Webley *et al*, 2001).

Monoclonal antibody A33 recognises a cell-surface antigen that is expressed on ∼95% of colon cancers (Garin-Chesa *et al*, 1996). In clinical trials, radiolabelled A33 localised specifically to colon cancer cells, where it was retained for several weeks while clearing within days from normal colon (Welt *et al*, 1996).

Cytosine deaminase (CD) converts 5-fluorocytosine (5-FC) into 5-fluorouracil (5-FU) and has been empolyed in ADEPT (Wallace *et al*, 1994).

Recombinant fusion constructs should overcome the problems of chemical antibody–enzyme conjugation including inhomogeneous products and large protein size. Several recombinant constructs based on F(ab) and F(ab′)_2_ fragments have been described. Constructs based on single-chain variable fragments (scFv) may have favourable diffusion characteristics in solid tumours, but few descriptions of this approach have been published (Bhatia *et al*, 2000). Here, we report on a new ADEPT concept based on the A33 antigen and recombinant scFv–CD constructs.

## MATERIALS AND METHODS

A33scFv (Rader *et al*, 2000) and CD (Austin and Huber, 1993) cDNA were PCR-amplified. Primers based on the published sequences were designed to remove start or stop codons and to add flanking restriction sites so that the DNA could be inserted into the pET 25 expression vector (Novagen, Madison, WI, USA) both directly and downstream of the inserted A33scFv DNA, so that the orientation of the fusion protein was 5′-A33scFv-CD-3′ (vector map available upon request).

A33scFv, CD, and A33scFv-CD and the control construct A33scFv-GFP were expressed by a T7-RNA polymerase-controlled bacterial system using BL21 *Escherichia coli λ*DE3 lysogens (Novagen, Madison, WI, USA) at 37°C with IPTG induction at an OD_600 nm_ of 0.5–0.7. Inclusion bodies were retrieved from cell pellets and solubilised using BugBuster™ reagent with 0.3 *μ*l ml^−1^ Benzonase and Novagen Refolding Kit (both: Novagen, Madison, WI, USA) according to the manufacturer's instructions. Utilizing a C-terminal histidin tag, the protein was purified on sepharose-bound cobalt (Clontech) with imidazole elution.

Plasmon surface resonance assays were performed as described (Catimel *et al*, 1997) with A33 antigen-coated Biosensor chips. After 400 s, sample flow was replaced by buffer solution. The relative refraction at 600 s was compared with buffer flow and positive controls.

Catalytic activity of cytosine deaminase was determined as described (Austin and Huber, 1993).

For cytotoxicity assays, LIM 1215 or HT 29 tumour cells (Ludwig Institute for Cancer Research cell bank) were incubated on 96-well plates to reach 25–33% surface density. Fusion protein or control was added for 60 min, preceded by 90 min of incubation with “A33scFv-GFP” or hu3S193 IgG (1 mg ml^−1^) in blocking experiments. After washing, cells were incubated with prodrug or control for 48 h, washed and grown in medium for 72 h, followed by 3 h in 0.5 mg ml^−1^ MTT-solution, DMSO-lysis and photometry at 595 nm.

## RESULTS

### Protein expression and activity

Fusion proteins were expressed as inclusion bodies with a final culture yield of about 100 μg l^−1^. With metal affinity chromatography purity was >95% by SDS–PAGE.

Calculated from the molar extinction coefficient of 1.038 mM^−1^ for 5-FU, the catalytic activity was 2.5 *μ*M min^−1^ for recombinant CD and 0.8 *μ*Mmin^−1^ for the A33scFv–CD fusion protein.

**Figure 1:**
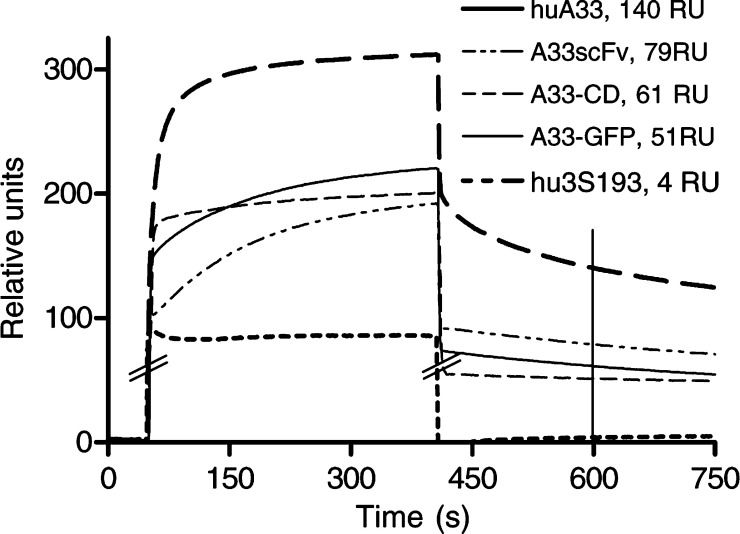
Surface Plasmon resonance. Association and dissociation curves of A33 antibody preparations on an A33 antigen-coated biochip. The chip was exposed to either the complete huA33 IgG antibody (huA33 IgG), the rabbit-derived single chain fragment (A33scFv) or inclusion body preparations of the fusion proteins of A33scFv with either cytosine deaminase (A33scFv–CD IB) or green fluorescent protein (A33scFv–GFP IB). At 400 s, antibody flow was stopped and the chip rinsed with buffer solution. Protein binding is measured by the refraction of a light beam and expressed in relative units (RU) over time. The 600 s time point and the relative units at this point are indicated as approximate correlates of affinity.

In surface plasmon resonance, all A33 preparations, but not the 3S193 control, displayed typical association and dissociation curves. Univalent A33scFv showed about half the binding activity of divalent of huA33 IgG (Figure 1

), and A33scFv–CD had slightly less binding activity than A33scFv.

### ADEPT system *in vitro*

The antigen binding and enzymatic activity of the A33scFv–CD fusion protein was assessed in cytotoxicity assays using complete ADEPT system. The cytotoxicities of 5-FC and 5-FU showed no significant differences between the colon cancer cell lines LIM1215 (A33+) and HT29 (A33–) with an IC_50_ of about 30 mM for 5-FC and 0.3–0.03 mM for 5-FU (*P*<0.05 for 5-FC *vs* 5-FU, no significant difference between cell lines).

**Figure 2:**
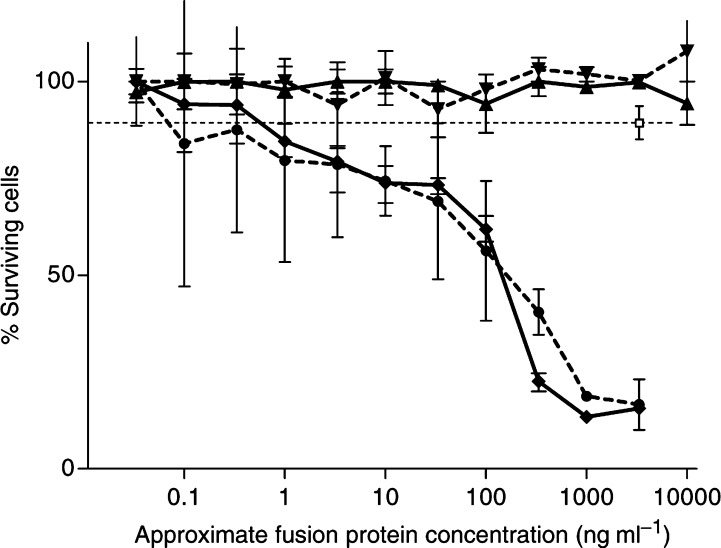
A33scFv–CD-mediated cytotoxicity on A33 antigen-positive *vs* negative cells: LIM1215 cells and HT29 cells were incubated with a dilution series of A33scFv–CD fusion protein and, after washing, with the 5-FC prodrug. Survival was measured by the MTT method as described. A33scFv–CD fusion protein from two different preparations was used on HT29 cells (▴ and ▾) and on LIM1215 cells (• and ⧫). As a control, a single, high concentration of A33scFv–GFP (□) was used instead of A33scFv–CD. Mean and s.d. of triplicate samples.

The complete ADEPT system was tested by incubating these two cell lines first with a serial dilution of A33scFv–CD and then, after washing, with the 5-FC prodrug at a fixed concentration. In this assay, crude and purified A33scFv–CD had a dose-dependant cytotoxic effect on A33-positive LIM1215 cells (IC_50_∼150 ng ml^−1^), but not on A33-negative cells (P=0.001 in Wilcoxon rank test). No cytotoxicity was observed with the A33scFv–GFP control (Figure 2

).

**Figure 3:**
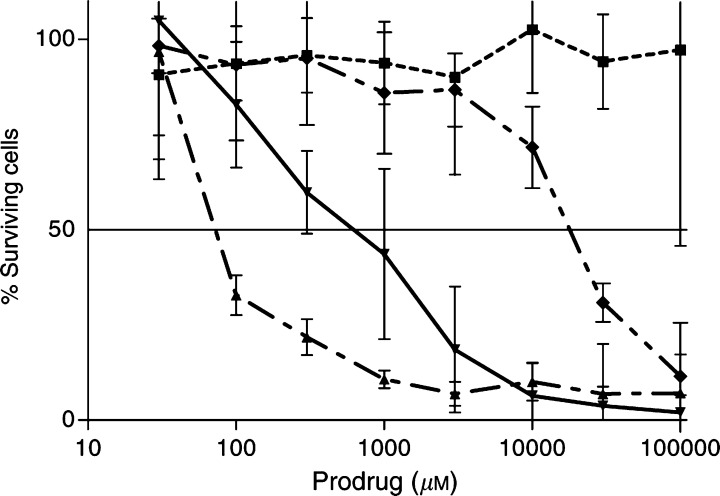
MTT cytotoxicity blocking assay. As a negative control, A33scFv–CD was used without subsequent prodrug incubation (▪), and 5-FU alone served as positive control (▴). In the complete ADEPT assay with subsequent 5-FC incubation as described in the text, cells were preincubated either with the “A33scFv-GFP” antibody (⧫) or with hu3S193 as an isotypic control antibody (▾) for 1 h before the fusion protein was added. Mean and s.d. of triplicate samples.

Without subsequent prodrug incubation, even the highest concentration of fusion protein tested had no cytotoxic effect on A33-positive LIM1215 cells (Figure 3

). When binding of A33scFv–CD was blocked by preincubation with “A33scFv-GFP”, subsequent 5-FC incubation showed reduced cytotoxicity (IC_50_, ∼30 mM) compared to wells containing the irrelevant isotype control antibody hu3S193 (IC_50_, <1 mM, P<0.01).

## DISCUSSION

Two major obstacles have hampered the progress of ADEPT: the needs for specific, accessible antigens and for chemically stable and defined antibody–enzyme constructs of suitable molecular size. The ADEPT system introduced here is novel regarding the targeted antigen and the use of a recombinant scFv-based CD construct.

Incubation of A33-positive tumour cells with this construct increased 5-FC toxicity by about 300-fold, which was selectively blocked by preincubation with “A33scFv-GFP”, demonstrating antigen specificity. Neither A33scFv–CD without 5-FC nor a control construct with 5-FC inhibited cell growth, showing that specific enzymatic conversion was necessary for cytotoxicity. Together, these results demonstrate dual (i.e antibody and enzyme) specificity of the construct and functioning of this ADEPT system *in vitro*.

For ADEPT, it is important that CD does not naturally occur in mammalians, making the enzyme construct the exclusive source of prodrug activation, while allogenic immunogenicity can be addressed by polyethylene-glycol conjugation with preserved A33 binding (Deckert *et al*, 2000).

Only recently has the homohexameric structure of bacterial CD been resolved (Ireton *et al*, 2002). When the described construct showed effective dual function, either its monomer has catalytic activity, or it can form oligomers in solution or after antigen binding. While the published structure supports monomer activity, both hypotheses would explain the lower catalytic activity of A33scFv–CD compared to enzyme alone.
